# Electrocatalysis of Oxygen Reduction Reaction on Shape-Controlled Pt and Pd Nanoparticles—Importance of Surface Cleanliness and Reconstruction

**DOI:** 10.3389/fchem.2019.00648

**Published:** 2019-10-04

**Authors:** Ruttala Devivaraprasad, Naresh Nalajala, Bapi Bera, Manoj Neergat

**Affiliations:** ^1^Department of Energy Science and Engineering, Indian Institute of Technology Bombay, Mumbai, India; ^2^National Chemical Laboratory, Catalysis Division, Pune, India

**Keywords:** adsorption, dissolution, oxygen reduction reaction, platinum, palladium, reconstruction, shape-control, surface cleaning

## Abstract

Shape-controlled precious metal nanoparticles have attracted significant research interest in the recent past due to their fundamental and scientific importance. Because of their crystallographic-orientation-dependent properties, these metal nanoparticles have tremendous implications in electrocatalysis. This review aims to discuss the strategies for synthesis of shape-controlled platinum (Pt) and palladium (Pd) nanoparticles and procedures for the surfactant removal, without compromising their surface structural integrity. In particular, the electrocatalysis of oxygen reduction reaction (ORR) on shape-controlled nanoparticles (Pt and Pd) is discussed and the results are analyzed in the context of that reported with single crystal electrodes. Accepted theories on the stability of precious metal nanoparticle surfaces under electrochemical conditions are revisited. Dissolution, reconstruction, and comprehensive views on the factors that contribute to the loss of electrochemically active surface area (ESA) of nanoparticles leading to an inevitable decrease in ORR activity are presented. The contribution of adsorbed electrolyte anions, *in-situ* generated adsorbates and contaminants toward the ESA reduction are also discussed. Methods for the revival of activity of surfaces contaminated with adsorbed impurities without perturbing the surface structure and its implications to electrocatalysis are reviewed.

## Introduction

Fuel cell, an electrochemical device, converts chemical energy to electrical energy. In low temperature polymer electrolyte membrane fuel cell (PEMFC), the electrodes are generally based on carbon-supported platinum (Pt) nanoparticles. In a cell, protons produced at the anode from the fuel oxidation reaction are transported through the electrolyte membrane (usually, Nafion®–a sulphonated tetrafluorethylene copolymer) to the cathode, wherein oxygen reduction reaction (ORR) takes place. The two reactions set up a potential difference between the electrodes and it drives the electrons through the external circuit. However, Pt-based precious metal catalysts are prohibitively expensive for the widespread use of PEMFCs. Therefore, it is imperative to reduce either the Pt content and improve its catalytic activity or to replace it completely with cheaper alternatives at the cathode in fuel cells.

Pd is considered to be a promising alternative to Pt, perhaps because of their similar crystal structure (face centered cubic) and electronic configuration (d-transition metals) (Mittermeier et al., 2017). Most importantly, Pd is situated at the peak of the volcano plot of ORR activity, adjacent to Pt (Nørskov et al., 2004). Though, the electrochemical stability of Pd in acidic conditions is relatively poor as compared to Pt, it is widely reported for direct formic acid fuel cells and alkaline fuel cells, which are fuelled with ethanol and propanol (Antolini, 2009; Shao, 2011; Shao et al., 2011). Pd and its alloys with transition metals are also widely investigated recently as electrocatalysts for ORR. Therefore, it is imperative to study its size, and shape-dependent electrochemical properties.

The kinetics of electrochemical reactions depends on the exposed crystallographic planes and the type of exposed active sites on the catalyst surface. Thus, many research groups investigated electrooxidation of organic molecules (formic acid, carbon monoxide, methanol etc.), hydrogen evolution, and oxygen reduction on low-index crystallographic planes of Pt and Pd (Housmans and Koper, 2003; Hoshi et al., 2006; Solla-Gullón et al. 2008; Grozovski et al., 2010; Farias et al., 2014). In particular, the kinetics of ORR was found to be dependent on the exposed crystallographic planes ({hkl}) as reported with single crystal Pt and Pd surfaces and on electronic factors decided by the surface, subsurface and bulk compositions (Marković et al., 1994, 1995, 1997; Stamenkovic et al., 2002, 2006; Kondo et al., 2009). Furthermore, the kinetics of ORR on such well-defined surfaces is known to be influenced by the adsorption of anions such as OH^−^, ${\text{ClO}}_{4}^{-}$, and ${\text{HSO}}_{4}^{-}$ ions from NaOH, HClO_4_, and H_2_SO_4_ electrolyte medium, respectively.

The findings that established crystallographic orientation dependent ORR activity with well-defined surfaces have rarely been translated to bulk practical electrocatalysts. Shape-controlled nanoparticles surface terminated with facets of most active sites can be used to take advantage of the facet-dependence of electrochemical reaction. Therefore, many research groups shifted their focus toward shape-controlled Pt and Pd nanoparticles, where, one can exploit the active site dependence of ORR by populating the selective crystallographic orientations. The key to achieve shape-controlled synthesis involves enhancing or inhibiting the growth along certain crystallographic directions, either by introducing surface energy differences in the seeds of the particles, or by preferential bonding of surfactants. The strong binding of the stabilizing agent/surfactant on the catalyst surface severely hampers the catalytic activity. Therefore, removal of the surface-adsorbed stabilizing agents after the synthesis (cleaning of the nanoparticles) is a crucial requirement, especially in the context of electrocatalytic applications.

The other issues debated over the last few decades are the stability and durability of metal nanoparticles, in particular that of Pt and Pd nanoparticle surfaces in electrochemical environment (or in the presence of adsorbates) (Clavilier and Armand, 1986; Wagner and Ross, 1988; Kolb, 1995). Previous studies have shown that the loss of electrochemical surface area (ESA) of metal nanoparticles limits the long-term performance (durability) of PEMFCs (Ferreira et al., 2005; Bi et al., 2007; Shao-Horn et al., 2007; Tripkovic et al., 2008). Two processes are believed to contribute significantly to the ESA loss of Pt nanoparticles in PEMFCs: (i) coarsening of Pt nanoparticles by the dissolution and redeposition of particles (Ostwald Ripening) and (ii) loss of Pt mass from the cathode (dissolution) (Ferreira et al., 2005; Yasuda et al., 2006; Bi et al., 2007; Shao-Horn et al., 2007). Before investigating with the real/bulk catalysts used in fuel cells, researchers studied reconstruction of the well-defined surfaces in contact with an aqueous electrolyte medium. Nevertheless, attempts to translate the findings from studies on single crystal electrodes to bulk practical electrocatalysts have met with several challenges.

Therefore, this review aims to briefly discuss the shape-controlled synthesis of Pt and Pd nanoparticles, their cleaning methods, characterization and electrocatalytic activity toward ORR. The synthesis and cleaning of shape-controlled Pt and Pd have several commonalities; in this context, we have included both. This is rather a fundamental review and device level characteristics are not discussed. The effect of geometric and electronic factors on ORR with shape-controlled nanoparticles is discussed in detail and compared with that of single crystal surfaces. Comprehensive views are presented regarding the stability, durability and non-recoverable ESA loss of Pt shape-controlled nanoparticles under electrochemical conditions; their implications in electrocatalytic applications are also presented.

## Synthesis

### Shape-Controlled Pt Nanoparticles

Over the past few decades, shape-controlled metal nanoparticles [e.g., platinum cuboctahedral (Pt-CO), platinum nanocube (Pt-NC), platinum tetrahedral (Pt-TD), and platinum polycrystalline (Pt-PC)] were synthesized by various synthesis protocols (Tao et al., 2008; Xia et al., 2009; Lee et al., 2012a,b; Zhou and Li, 2012; Quan et al., 2013) and several excellent reviews and reports are available on these methods (Burda et al., 2005; Guo and Wang, 2011; Chen et al., 2012; Gou et al., 2013; Zhang et al., 2013; Weiner et al., 2015; Xia et al., 2015, 2017; Gilroy et al., 2018). Herein, we have briefly discussed synthesis methods (see the Table 1) considering the applications of shape-controlled nanoparticles in the electrocatalysis of ORR. Controlling the nucleation processes during the synthesis is the key step in achieving mono-disperse shape-controlled nanoparticles. The overgrowth step can be controlled by (i) reducing agents/foreign metal ions (ii) organic capping agents, and (iii) inorganic capping agents (Lee, 2014).

**Table 1:** Description of synthesis of shaped-controlled Pt and Pd NPs.

**Precursor**	**Reductant**	**Surfactant**	**Additives**	**Conditions**	**Shapes (both Pt and Pd)**	**Cleaning procedure**	**References**
Pt(acac)_2_	N_2_H_4_·H_2_O	Oleic acid oleylamine	Fe(CO)_5_	200°C	Nanocubes and Nanospheres	Wash with ethanol and hexane	Naskar et al., 2017
H_2_PtCl_6_·6H_2_O, Pt(acac)_2_	EG, HCOOH	PVP, CTAB	HClO_4_	180°C	Nanocube	Wash with ethanol, hexane and DI water	Arán-Ais et al., 2018
H_2_PtCl_6_	NaBH_4_	–	HCl	Room temperature	Nanocube	Acetone and heptane at the ratio of 1:1 and DI water	Ehrenburg et al., 2017
H_2_PtCl_6_·6 H_2_O	EG	PVP	–	Room temperature	Nanocube	Wash with ethanol and hexane	Safo and Oezaslan, 2017
PdCl_2_, H_2_PtCl_6_	Glucose, L-ascorbic acid, EG	PVP, CTAB, SDS, oleylamine, oleicacid	–	–	–	–	Liu et al., 2017
K_2_PtCl_4_ H_2_PtCl_6_•6H_2_O	NaBH_4_		HCl	Room temperature	Octahedral, cubic, quasi-spherical	DI water	Perales-Rondón et al., 2017
K_2_PtCl_4_	–	Sodium polyacrylate	–	Room temperature	Cubic and octahedral	DI water and NaOH	Farias et al., 2017
K_2_PtCl_4_	–	Amine terminated polyamidoamine dendrimers (PAMAM)	Chitosan (CS)	180°C	Flower-like spherical	DI water	Wang et al., 2016
H_2_PtCl_6_	FeCl_3_	–	FeCl_3_	Two step temperature 100 and 180°C	Nanopeanut	Washing in ethanol	Zhang et al., 2016
Pt(acac)_2_ Na_2_PdCl_4_	L- ascorbic acid	PVP, oleylamine	NaBr	250 and 100°C	Cubic	Acetone, ethanol, and DI water	Lee et al., 2015
Pt(acac)_2_		Oleylamine and oleic acid		240°C	Quasi-spherical and cubic	Hexane and ethano MeOH + NaOH/acetone was repeated at least three times, DI water	Arán-Ais et al., 2015a,b
H_2_PtCl_6_	NaBH_4_	–	HCl	Room temperature	Cubic	Acetone water mixtures	Martinez-Rodriguez et al., 2014
K_2_PtCl_4_ or H_2_PtCl_6_	NaBH4	–	–	Room temperature	Cubic and octahedral	NaOH pellets/DI water	Farias et al., 2017
Pt(acac)_2_ K_2_PtCl_6_ K_2_PtCl_4_	Formic acid	–	PtBr_2_	120°C	Cubic, Truncated octahedral	Ethanol–water	Gumeci et al., 2014
Pt(acac)_2_	Mn_2_(CO)_10_	Oleylamine, oleic acid	–	160–240°C	Cubes, Octahedra, Truncated Cubes and Icosahedra, Cuboctahedra, Spheres, Tetrapods, Star-like Octapods	Wash with hexane	Kang et al., 2013
Pt(acac)_2_	–	Oleylamine, oleic acid	KBr	160–240°C	Cubes, Truncated Cubes, polyhedral	–	Guo and Wang, 2011
K_2_PtCl_4_	NaBH_4_ Ethylene glycol	CTAB	–	–	Cubic, octahedral/tetrahedral, cuboctahedrons/truncated octahedrons	–	Coutanceau et al., 2012
H_2_PtCl_6_	NaBH_4_	Sodium polyacrylate	–	Room temperature	Cubic, octahedral, tetrahedral shape, truncated octahedral and tetrahedral	DI water/NaOH	Vidal-Iglesias et al., 2012
PdCl_2_	L-ascorbic acid, NaBH_4_	CTAB, CTAC	NaI, KI, NaBr	95°C	Cubic and Octahedral	DI water	Sneed et al., 2012
H_2_PtCl_6_·xH_2_O	NaBH_4_, ascorbic acid	–	–	Room temperature	cubic, multipods, bipyramid	DI water	Ruan et al., 2011
Na_2_PdCl_4_	L-Ascorbic acid	Poly(vinyl pyrrolidone) (PVP)	KBr and KCl	80°C, 3 h	Cubes, Bars	Washed with water for 10 times	Jin et al., 2011a
K_2_PdBr_4_, Na_2_PdCl_4_	Sodium adsorbate (NaAsc)	PVP	KBr	25°C, 3 h	Concave nanocubes	–	Vara and Xia, 2018
PdCl_2_ · (CH_3_CN)_2_	H_2_ gas (5 bar)	Oleylamine and oleic acid	–	25°C, 1 h	Tripod	Methanol followed by dichloromethane and toluene.	Watt et al., 2009
H_2_PdCl_4_	L-Ascorbic acid	Cetyltrimethylammonium bromide (CTAB)	KI	30–80°C, 40 min−1 h	Cube, Octahedron, and RD	–	Niu et al., 2010
Pd(acac)_2_	Formaldehyde	Oleylamine	–	100°C, 8 h	Icosahedron, decahedron, octahedron, tetrahedron, and triangular plates	Acetic acid treatment at 70°C for 10 h	Mazumder and Sun, 2009; Niu et al., 2011
Na_2_PdCl_4_ and Pd(acac)_2_	Tetraethylene glycol (TTEG)	PVP	–	140°C, 1 h	Cuboctahedral, octahedrons, and tetrahedrons	Washing with Acetone and Water for three times	Wang et al., 2013
Na_2_PdCl_4_	Diethylene glycol	PVP	Na_2_SO_4_ and HCl	105°C, 3 h	Decahedra and Icosahedra	Once with acetone and then twice with DI water	Huang et al., 2014
PdCl_2_	Electrochemical method	–	–	25°C	Tetrahexhedral		Tian et al., 2010
Na_2_PdCl_4_	L-Ascorbic acid	PVP	KBr	60°C, 3 h	Concave nanocube	Washing with water for three times	Jin et al., 2011b
PdCl_2_	Ascorbic acid	Cetylpyridinium chloride	HCl	80°C, 1 h	Nanocube, cuboctahedra, octahedra	Several times with ethanol	Zhang et al., 2014
Na_2_PdCl_4_	Ascorbic acid, Diethylene glycol	PVP	NaI, FeCl_3_, Na_2_SO_4_, NaCl	104°C, 3 h	Decahedra, nanorod	Three times with acetone and water	Ruditskiy et al., 2017
PdCl_2_	Ascorbic acid, sodium borohydride,	CTAB (NaBr), and (CTAC)	HCl	30°C, 10 min	Nanocube, concave nanocube, nanoflowere		Sreedhala et al., 2014
PdCl_2_	Ascorbic acid	CTAB	(CuSO_4_), (CuCl_2_), (Cu(NO_3_)_2_), HCl	95°C, 12 h	Nanocubes, concave nanocubes	Two times with water	Niu et al., 2014
PdCl_2_	Ascorbic acid	CTAB, CTAC		30°C, 7 h	Concave nanocubes	Electrochemical cleaning	Zhang et al., 2011
PdCl_2_	Ascorbic acid	CTAC	KBr, KI	30–60°C, 30 min	Nanocubes, concave nanocubes, octahedral, truncated octahedral, cuboctahedral	Washing with water	Liu et al., 2015
K_2_PdCl_4_	Ascorbic acid	CTAB		35–40°C	Concave nanocubes	Washing with water	Xie et al., 2015
Na_2_PdCl_4_	Ascorbic acid	PVP	KBr, KCl	25°C, 3 h under UV-visible irradiation	Truncated cubes, cubes	Acetic acid washing	Vara et al., 2017
Na_2_PdCl_4_	Ascorbic acid, Diethylene glycol, Ethylene glycol, Formaldehyde, Citric acid	PVP	NaI, KBr, KCl, HCl	80°C, 3 h 160°C, 1 h	Nanowires, nanocubes	One time with acetone, two times with water	Peng et al., 2016
H_2_PdCl_4_	Carbon monoxide (CO)	–	CO	Ambient temperature, 30 min	Nanosheets	Organic ligand free synthesis	Li et al., 2013
H_2_PdCl_4_	Ascorbic acid	CTAB	KI	95°C, 30 min	Rhombic dodecahedral, Cubic	Repeated CO adsorption replacement and anodic stripping	Zhang et al., 2012
K_2_PdCl_4_	NaBH_4_	CTAB	–	30°C, 2 h	Pyramid, pentatwinned nanorods, cube, icosahedra	–	Bisson et al., 2009
Na_2_PdCl_4_ PdCl_2_	Ascorbic acid	Cinchonidin, S-proline CTAB, PVP	HCl	50–95°C, 20 min−3 h	Nanocube, nanodendrite	Washed by water once and methanol twice	Gao et al., 2019
K_2_PdCl_4_	NaBH_4_	Dodecanethiol (DDT), oleylamine (OAm), and PVP	–	60–90°C, 3 h	Polycrystalline	Thermal treatment Chemical treatments	Collins et al., 2018
H_2_PdCl_4_	L-Ascorbic acid	Dioctadecyldimethylammonium chloride (DODAC)	–	95°C, 30 min	Nanowires	UV/Ozone treatment for 2 h	Xu et al., 2016

Using Ag ions, gaseous CO, or halide ions as foreign additives, Pt nanoparticles with uniform shape and size were synthesized (Lee, 2014). Excess amount of Ag ions was reported to convert cubes to cuboctahedra to octahedra (Lee, 2014). Halide ions such as I^−^ and Cl^−^ induced formation of cubic and octahedral Pt nanoparticles, respectively (Lee, 2014). However, removal of these additives is a daunting task and thus the ORR activity obtained from such Pt nanoparticles were significantly lower than that of the commercial Pt-PC catalyst from Jhonson Mattey.

A short review by Mourdikoudis and Liz-Marzán (2013) reported on all the important aspects of the suitability and the versatility of oleylamine/oleic acid (OAm/OAc) reaction medium for the synthesis of a broad range of nanoscale materials. Since the primary focus of this review is something different, extensive details of the synthesis is avoided.

Essentially, it was established that OAm and OAc work in combination to produce several different shaped nanoparticles under specific experimental conditions. Importantly, it was identified that the composition of OAm/OAc plays a crucial role in deciding the final morphology of nanoparticles. OAm is long-chain primary alkylamine, which acts as electron donor, exhibits basicity and affinity to metals through their NH_2_ functional groups, and the resulting morphology and crystallinity of the produced nanoparticles can be significantly different based on the solvent, surfactant and reducing agent. Moreover, OAm is a liquid at room temperature, which may simplify the washing procedures, following the chemical synthesis of nanoparticles. Other advantages of the use of OAm are its easy removal via centrifugation, high boiling point, low cost and tendency to form metal-OAm complexes at intermediate temperatures, so that it can be controllably decomposed to produce nanoparticles. On the other hand, OAc with carboxylic acid functional groups induces the structural changes in the nanoparticles. The composition, size and shape of the obtained nanoparticles can be tuned by careful choice of additional reaction parameters, depending on the system under study. Thus, two of the most popular organic reagents used for the shape-control are oleic acid and oleylamine (Chen et al., 2009). Organic reagents help prevent aggregation of nanoparticles during the synthesis and a variety of shapes (cubes, tripods, octapods, prisms, rods etc.) of Pt nanoparticles were synthesized. In particular, amine-containing capping agents are widely used in the shape-controlled synthesis because of the strong interaction between nitrogen and Pt surface. For e.g., using adamantane carboxylic acid (ACA) and hexadecylamine (HDA) as capping agents, Pt cubes, tripods and multipods were prepared (Chen et al., 2009).

**Figure 1:**
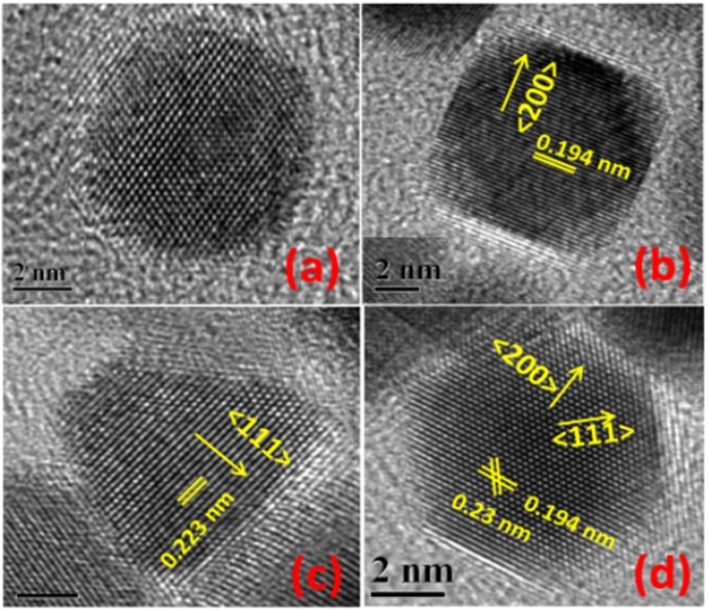
HR-TEM images of single Pt-PC **(a)**, Pt-NC **(b)**, Pt-TD **(c)**, and Pt-CO **(d)** nanoparticles. Reproduced and modified with permission (Devivaraprasad et al., 2014). Copyright 2014, American Chemical Society (ACS).

Shape-controlled Pt nanoparticles were also synthesized using various other surfactants and capping agents (citric acid, polyvinylpyrrolidone (PVP), cetyl trimethylammonium bromide (CTAB), ascorbic acid, etc.) (Chen et al., 2009; Peng and Yang, 2009; Chun-Jiang and Schuth, 2011). Most importantly, Ahmadi et al. (1996) reported the synthesis of cubic, tetrahedral and cuboctahedral Pt nanoparticles, surface-terminated with low-index facets. For e.g., cubic shape is bound by {100} planes, cuboctahedron by a mix of {111} and {100} planes, and icosahedron by {111} planes. Ahmadi et al. (1996) reported chemical reduction of K_2_PtCl_4_/H_2_PtCl_6_ of specific composition by H_2_ in the presence of polyacrylate (PAA) to produce shape-controlled Pt nanoparticles. Figure 1 shows the HRTEM images of shape-controlled Pt nanoparticles obtained with one such method. Because of the easinesss with the experimental conditions and the PAA surfactant removal method, later on, many researchers employed similar synthesis procedures to obtain various Pt shape-controlled nanoparticles by changing the respective precursors, reduction conditions with H_2_, and the concentration of the PAA (Ahmadi et al., 1996; Solla-Gullón et al., 2003, 2008; Chen et al., 2009). Henceforth, in electrocatalysis of ORR, emphasis has been on enhancing the activity and durability of shape-controlled nanoparticles, and in understanding their relevance toward the practical electrocatalysts employed in fuel cell application.

### Shape-Controlled Pd Nanoparticles

Different morphologies of Pd were obtained by changing the Pd precursor concentration during the electrodeposition, for e.g., Pd nanorods with dominant (110) facets were obtained from 3 × 10^−4^ M PdCl_2_ and Pd nanoparticles with (100), and (111) facets and other disordered structures from 3 × 10^−5^ M PdCl_2_ (Xiao et al., 2009). The synthesis of Pd nanocubes of size ~30 nm was carried out using CTAB as structural control agent, ascorbic acid as reducing agent and H_2_PdCl_4_ as the metal precursor. Besides, Pd polycrystalline (Pd-PC) nanoparticles (~3 nm) were prepared using NaBH_4_ and sodium citrate as reducing agent and stabilizer, respectively (Erikson et al., 2011).

Seed-mediated synthesis is also a powerful strategy to produce colloidal metal nanocrystals. Under controlled experimental conditions, this approach enables reproducible synthesis of nanoparticles, minimizing wastage of reagents. It offers a facile method to engineer the surface of the nanoparticle; in general, the facets of the end product replicates the surface structure of the initial seed. It allows to tune the surface of nanostructure using the capping agents present in the solution.

**Figure 2:**
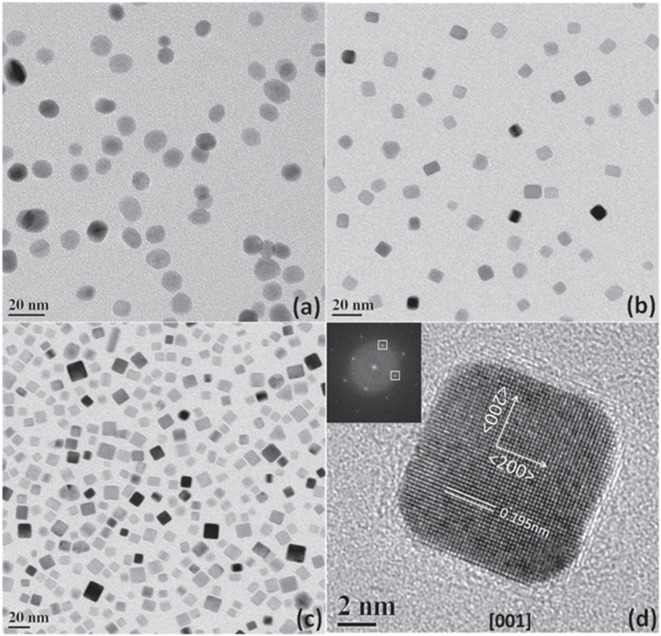
TEM images of Pd nanoparticles using Cl^−^ ions as capping agent under different atmospheres: **(a)** in ambient air; **(b)** in Ar; **(c)** in Ar at 80°C; and **(d)** the HRTEM image of selected nanoparticle; inset of **(c)** shows FFT of that nanoparticle. Reproduced with permission (Nalajala et al., 2016). Copyright 2016, Institute of Physics (IOP).

The preparation of shape-controlled metal nanoparticles using surfactant-free or weakly adsorbed capping ligands those can easily be removed is particularly attractive for electrocatalytic ORR. In this context, Cl^−^ ion mediated shape-control of Pd nanoparticles was achieved by changing concentrations of Cl^−^ ions, the synthesis temperature, and the reaction atmosphere (argon, air, and oxygen); it is possible to control the shape and size of Pd nanoparticles (Nalajala et al., 2016). The study emphasized that Cl^−^ ions can control the shape of Pd and can produce nanocubes of size ~8 nm from solution with dissolved oxygen content, as shown in Figure 2. Under appropriate experimental conditions, with the help of Cl^−^ ions as capping agent, various shapes of Pd such as nanocube, octahedral, multiple twinned particles (MTP), and nanorods were obtained. The shape-control of Pd using strongly adsorbing Br^−^ and I^−^ ions were also investigated and it was found that the dissolved oxygen could not affect the shape of Pd nanoparticles (Nalajala et al., 2016).

Nanosheets of different thickness (3 monolayers (ML), 5 ML, 8 ML) and of different edge length (ranging from ~120 to ~260 nm) were prepared using CO as a reducing as well as stabilizing ligand and oleylamine as solvent (Wang et al., 2019). For the first time, the hydride form of Pd nanocubes (PdH_x_ for x = 0, 0.43, 0.706) was established for ORR in O_2_-saturated 0.1 M HClO_4_ electrolyte. The proposed nanostructures were prepared using autoclave method with DMF as a solvent, PVP as a stabilizer, NaI as a capping agent and Pd(II) acetylacetonate as a precursor (Lu et al., 2017). The Pd-NCs dominant with (100) facets and rhombic dodecahedron dominant with (110) facets were prepared using an aqueous one-pot synthesis method. In the preparation of these nanostructures at 90°C, KI was used as capping agent and CTAB was the stabilizing agent at different concentrations (Tang et al., 2016).

The capping agent may simply be a liberated byproduct or it can also be intentionally added during a solution-phase synthesis to control the shape of a nanoparticle. Through its chemical interaction with a metal surface, the presence of a capping agent can change free energies of different crystallographic planes, and thus their relative growth rates. Generally speaking, the binding affinity of a capping agent can vary from one crystal facet to another. Such preferential capping can effectively hinder the growth of a particular facet, thus providing a means for controlling the relative surface areas of different facets.

For e.g., PVP, a polymeric capping agent, binds strongly to the {100} facets of Ag and Pd through oxygen atoms. This preferential capping can drive the addition of metal atoms to the other crystal facets when crystal seeds are suitably large. Thus, for single-crystal seeds terminated with only {111} and {100} facets, metal atoms will add preferentially to the poorly passivated {111} facets (Xia et al., 2009). In contrast, PVP, Br^−^, and citrate ions bind most strongly to {111} facets, thus favoring the formation of octahedrons, icosahedrons, and decahedrons. The use of capping agents to control the shape of a nanoparticles makes some facets thermodynamically more favorable by reducing their interfacial free energies through chemisorption. Though the capping agents play a vital role in controlling the shape of nanoparticles, a complete understanding of the mechanism of shape-control still remains elusive.

## Surfactant Removal Methods

### Shape-Controlled Pt Nanoparticles

Nanoparticles contaminated with impurities, from the reagents used for the synthesis (e.g., capping agents and surfactants), cannot be directly used as electrocatalysts. It is necessary to clean the nanoparticles before subjecting them to ORR studies because of the coverage of active sites with organic ligands those were used to control size, shape and composition. Such clean nanoparticles are important in deriving any conclusions related to surface orientation. Therefore, it is imperative to develop methods for the removal of impurities without disturbing the size, shape, composition, and most importantly the surface structure of target nanoparticles.

Literature is replete with procedures for the removal of organic impurities from the surface of the particle and that involve thermal annealing, UV-ozone irradiation, and acid wash (Vig, 1985; Aliaga et al., 2009; Crespo-Quesada et al., 2011; Long et al., 2011; Monzo et al., 2012; Niu and Li, 2014; Niu et al., 2014; Yang et al., 2014; Arán-Ais et al., 2015a). The UV/ozone cleaning procedure can remove impurities from the particle surfaces in air or in vacuum atmosphere at room temperature in less than a minute. The surface cleanliness of nanoparticles was evidenced by the Auger electron spectroscopy, Electron Spectroscopy for Chemical Analysis (ESCA), and Ion Scattering Spectroscopy/Secondary Ion Mass Spectrometry (ISS/SIMS) studies (Vig, 1985). Aliaga et al. (2009) reported the removal of PVP and TTAB from Pt nanoparticles by the UV-ozone cleaning method. The effectiveness of this method was confirmed by several analytical measurements including Sum Frequency Generation Vibrational Spectroscopy (SFGVS), X-ray Photoelectron Spectroscopy (XPS) and Diffuse Reflectance Infrared Fourier Transform Spectroscopy (DRIFTS) (Aliaga et al., 2009). The same morphological features that were obtained from TEM of the cleaned nanoparticles and that of the as-prepared counterparts demonstrated that the UV-ozone treatment did not affect the shape of the Pt nanoparticles.

Out of several PVP removal procedures studied, it was reported that heat treatment/thermal annealing at 300°C could cause structural destruction of Pt nanoparticles; the treated particles were synthesized using polyol method by the reduction of H_2_PtCl_6_ with AgNO_3_ and ethylene glycol (Long et al., 2011). Nevertheless, the ORR activity of such nanoparticles was shown to be inferior due to the nanoparticle aggregation. Monzo et al. (2012) reported an effective method for cleaning Pt nanoparticles capped with PVP, without affecting their surface structure. The cleaning of Pt nanoparticles involved mixing of colloidal solution of Pt nanoparticles with H_2_O_2_/H_2_SO_2_ of appropriate concentrations, followed by centrifugation for several times. The characteristic features corresponding to the presence of (111) ordered surface domains demonstrated the extent of cleanliness of Pt nanoparticles (Monzo et al., 2012). Using electrocatalytic ORR as a probe, the effectiveness of the methods for oleylamine removal from oleylamine-capped Pt nanoparticles was found to be in the order of thermal annealing at 185°C in air > acetic acid washing > UV-ozone irradiation (Niu et al., 2014).

Contrary to most of the published reports, Vidal-Iglesias et al. (2011) showed the adverse effect of UV/ozone treatment involved in the decontamination of shape-controlled Pt nanoparticles prepared with PAA-TTAB. With the help of electrochemical techniques, this group successfully demonstrated that the UV/ozone cleaning process keeps the shape intact and disturbs the surface structure. Such unintentional surface perturbations may alter the catalytic activity. They demonstrated the weakness of conventional TEM toward such changes in the surface structure while it does give evidence for the change in shape.

In addition to the methods discussed above, electrochemical procedures were also widely used for the removal of surfactants from the catalyst surface. Thus, Pt nanoparticles were cleaned by potential cycling (in the range of 0.05–1.0 V) in appropriate electrolytes. Reproducible voltammograms are indication of the cleanliness. However, this procedure causes reconstruction of the surface and more details on the same will be discussed in the following sections. Solla-Gullón et al. (2003) used a modified cleaning method in sulfuric acid solution, which involves holding the electrode at 0.03 V for 3 min. The electrode was subsequently subjected to potential cycling in the range of 0.04–0.4 V. After repeating the above-mentioned procedure three times, the cleanliness of the surface was confirmed through the systematic voltammetric analysis.

Although various cleaning protocols have been proposed and proved to be efficient enough, it is important to understand that, most of these cleaning procedures are specific to the removal of individual surfactant, stabilizer, or synthesis protocols employed; therefore, the search for universal procedure for the removal of surfactant still continues.

### Shape-Controlled Pd Nanoparticles

**Figure 3:**
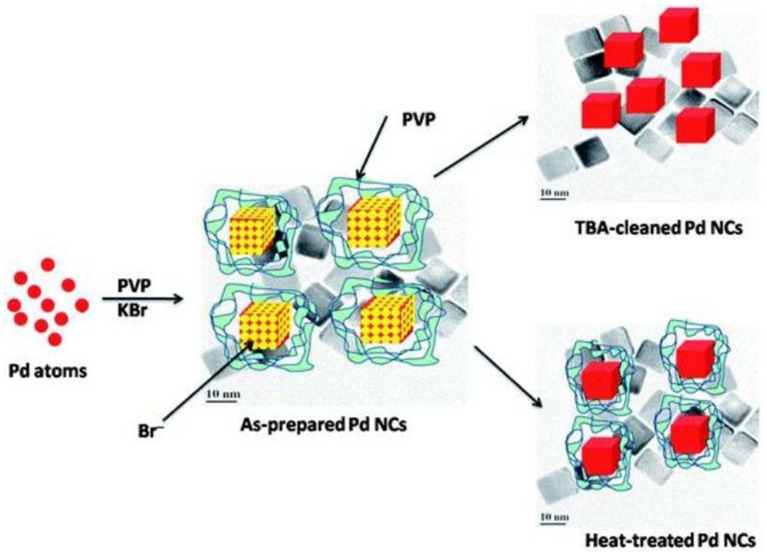
Scheme proposed for the removal of PVP and Br^−^ ions using TBA treatment and heat treatment. Reproduced with permission (Nalajala et al., 2013). Copyright 2013, Royal Society of Chemistry (RSC).

The removal of surfactant and capping agent was demonstrated with Pd-NCs synthesized by the solution phase method at 85°C in which PVP (stabilizer), L-Ascorbic acid (reducing agent), KBr (capping agent), and K_2_PdCl_4_ (precursor) were used. Voltammetric features of Pd were not observed from the as-prepared Pd-NCs and it demonstrated that the catalyst surface was completely contaminated with adsorbed impurities (residual PVP and Br^−^). Nalajala et al. (2013) reported a process for the removal of afore-mentioned impurities from Pd-NCs on treatment with tertiary butylamine (TBA). The cleanliness of Pd-NCs was confirmed by both physical and electrochemical characterizations. The sharp H_upd_ features and ORR voltammograms in argon-saturated and oxygen-saturated 0.1 M HClO_4_ solutions, respectively, demonstrated that the Pd-NCs were cleaned after TBA-treatment. Therefore, it was established that TBA could be a better solvent for the removal impurities from Pd-NCs, without affecting their surface structural integrity (Figure 3). TBA reacts with Br^−^ ions to form a quaternary ammonium salt that can easily dissolve in aqueous solution and thus cause removal of Br^−^ ions.

The same group further established processes for decontaminating stabilizer (PVP) and capping agents (citrates and halides), by exploiting the inherent property of Pd to form hydride (Nalajala et al., 2014). Thus, the exposure of as-prepared shape-controlled Pd nanoparticles to NaBH_4_ helps remove the adsorbed impurities, by weakening their interaction with the Pd metal hydride surface. The extent of cleaning after the treatment with NaBH_4_ was demonstrated by physical and electrochemical characterizations. Overall, weakly adsorbing reagents are preferred for shape-control of Pd nanoparticles because of the easy removal of impurities, without disrupting the surface structural integrity.

In order to remove the impurities from the Pd nanocubes and octahedral nanoparticle surface, steam treatment in a lab-made reactor was developed by Yang et al. (2018). The nanoparticles obtained after the steam treatment were intact with their shape and size. This steam cleaning process was reported to effectively remove adsorbed PVP from the Pd nanoparticles. Pd NCs prepared using PVP as stabilizing agent and NaI as capping agent were treated with ice cold NaBH_4_ for 30 min under vigorous stirring followed by washing with water-ethanol mixture. The basis for the removal of impurities was the formation of hydride, which in fact weakens the interaction between the impurities and metal surface, helping easy desorption from the surface (Lu et al., 2017). The Pd nanoparticles those were synthesized using PVP as stabilizer and KBr and citric acid as capping agents were subjected to non-destructive methods under electrochemical conditions, by holding the electrode consisting of thin layer of nanoparticles at −0.05 V vs. RHE for 60 seconds (Shao, 2011; Shao et al., 2011, 2013). After the synthesis, the prepared nanoparticles were treated with strong basic solution followed by the electrochemical oxidation of pre-adsorbed CO to produce clean nanoparticles (Erikson et al., 2012). Thermal annealing process at 300°C was implemented for the removal of surfactant (KI and CTAB) without disturbing the structural integrity of the Pd nanostructures (rhombic dodecahedral and cubic) (Tang et al., 2016). The cleaning of carbon-supported Pd-NCs of different sizes (~7, ~10, ~30 nm) was also performed by the addition of NaOH pellets followed by washing the mixture with DI water (Erikson et al., 2016).

## Structure Sensitivity of ORR With Single Crystal Electrodes and Shape-Controlled Metal Nanoparticles

### Case of Platinum

**Figure 4:**
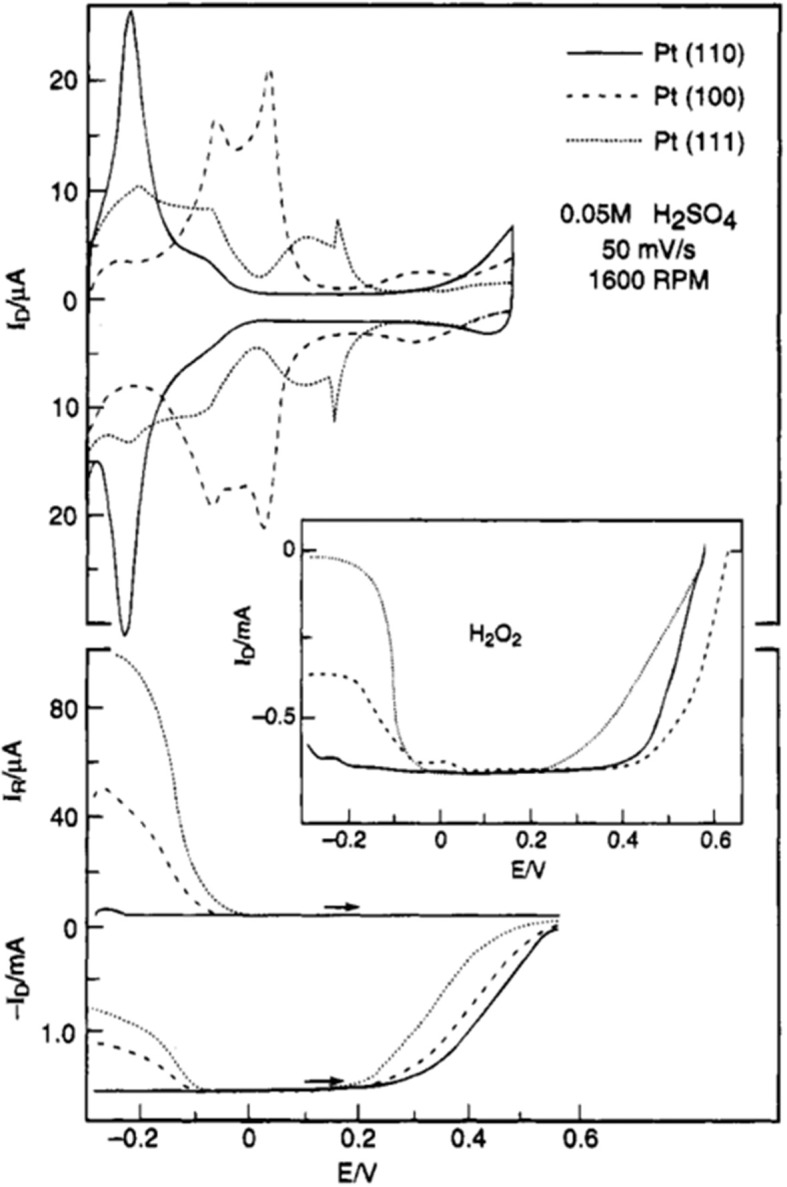
**Top:** CVs of Pt(hkl) in oxygen-free electrolyte in the RRDE assembly (fifth sweep). **Bottom:** ORR of Pt(hkl) in oxygen-saturated electrolyte (ring potential = 0.95 V). Insert: reduction of 1.2 × 10^−3^ M H_2_O_2_ on Pt(hkl**)** mounted in the RRDE assembly (0.05 M H_2_SO_4_, 50 mV/s, 1,600 rpm). Reproduced with permission (Marković et al., 1995). Copyright 1995, American Chemical Society (ACS).

It was established that the ORR activity of single crystal Pt electrodes is determined by the type of kinks, steps, terraces and sites on the catalyst surface (Marković et al., 1995; Macia et al., 2004; Rizo et al., 2013; Arán-Ais et al., 2016). Figure 4 shows the work reported by Marković et al. (1995), and the ORR activity of single crystal Pt electrode in 0.05 M H_2_SO_4_ is in the order of {110} > {100} > {111}, and that of peroxide formation is {111} > {100} > {110}. On the other hand, the activity orders in 0.1 M HClO_4_ electrolyte (Marković et al., 1994, 1997) and that in alkaline electrolyte (Marković et al., 1995; Rizo et al., 2013) were found to be different. The difference in ORR activity trends in various electrolytes was attributed to the inhibiting effect of solution anions on different sites (Marković et al., 1995). The relatively higher activity in alkaline media (as compared to that in acidic media) was explained on the basis of lower steric hindrance offered by OH^−^ ions (compared to complex oxy-anions, for e.g., ${\text{HSO}}_{4}^{-}$ and ${\text{CIO}}_{4}^{-}$ (Clavilier and Armand, 1986; Durst et al., 2012).

**Figure 5:**
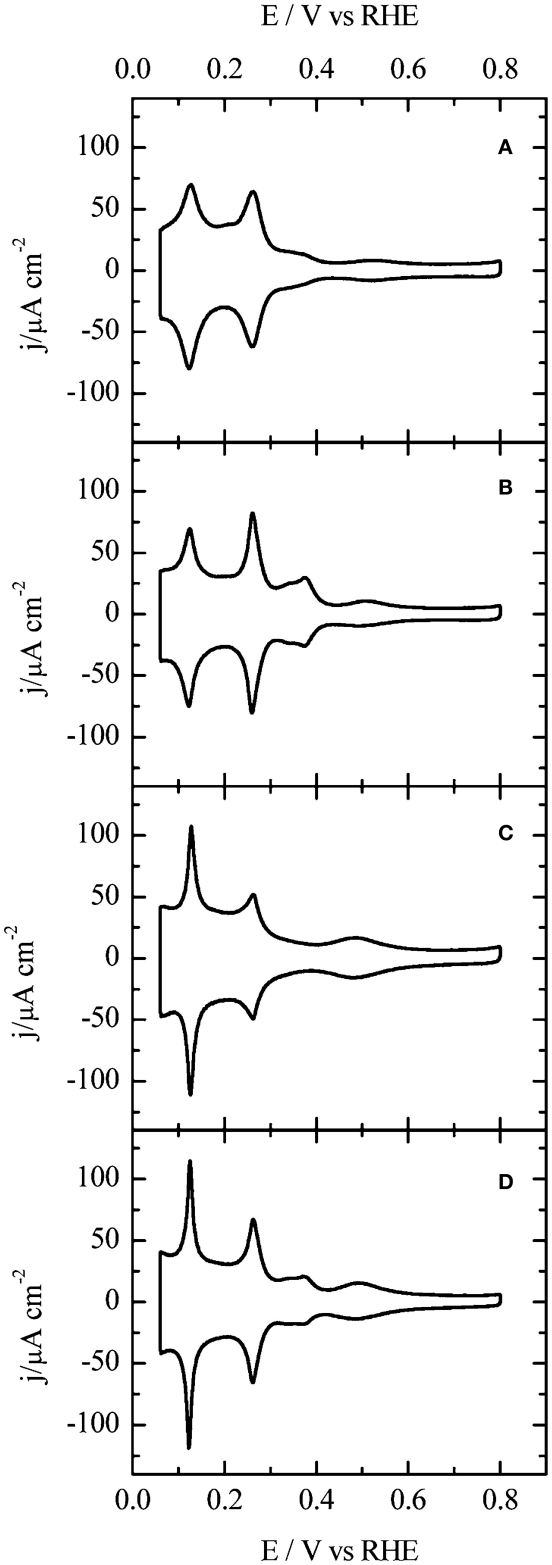
Voltammograms of **(A)** Pt-PC, and shape-controlled nanoparticles **(B)** Pt-NC, **(C)** Pt-TD, and **(D)** Pt-TO) recorded in 0.5 M H_2_SO_4_ at a scan rate of 50 mV s^−1^. Reproduced with permission (Vidal-Iglesias et al., 2012). Copyright 2012, American Chemical Society (ACS).

Electrochemical techniques offer detailed information on the surface structure of shape-controlled metal nanoparticles based on a large number of nanoparticles that are available on the electrode surface (Solla-Gullón et al., 2004, 2008; Chen et al., 2012; Farias et al., 2014; García-Cruz et al., 2019; Mayet et al., 2019). Voltammograms characteritic of low-index planes on the surface of Pt nanoparticles in both acidic and alkaline electrolytes were recently reported in the literature (Vidal-Iglesias et al., 2012; Devivaraprasad et al., 2014; Jukk et al., 2017). Such voltammograms of Pt shape-controlled nanoparticles recorded at a scan rate of 50 mV s^−1^ are shown in Figure 5 (Vidal-Iglesias et al., 2012). Since the shape-controlled nanoparticle surface is constructed with a combination of various facets of different H_ads/des_ energies, their specific H_upd_ features appear at different potentials. But, the potential ranges of the H_upd_ from low-index planes on the Pt surface overlap, despite being structure-sensitive. The distinct and unique voltammetric features in strongly adsorbing electrolytes are the fingerprint of surface-cleaned shape-controlled nanoparticles (Vidal-Iglesias et al., 2012). Overall adsorption charge obtained corresponds to the number of active sites and it is a measure of ESA. As with single crystal surfaces, the sharp and characteristic voltammetric profiles can be used to characterize the surface structure (ordered domains, steps, terrreses, and basal planes) and to qualitatively assess the surface faceting of various planes on Pt shape-controlled nanoparticles. An additional advantage is that these measurements are performed in solution, i.e., the environment in which electrochemical reactions occur and it is based on a large number of nanoparticles that are available on the electrode surface. In fact, surface atoms can undergo reconstruction and impurities modify the surface composition, and both can modify surface reactivity and have to be monitored. Therefore, it is imperative to investigate the reconstruction and dissolution of the shape-controlled nanoparticle surfaces in relevant electrolytes (electrochemical condition) to establish their stability prior to that of the reaction activity and its order. Thus, in acidic and alkaline electrolytes, the upper limit of the potential was restricted to 0.8 and 0.9 V, repectively. Cycling to potential above 1.0 V leads to reconstruction of the surface and as a result loss of characteristic voltammetric features of the faceted surface of the shape-controlled nanoparticle (Devivaraprasad et al., 2014).

Using conventional voltammetric analysis, it is hard to differentiate the surface faceting with complex coordinations like terrace, steps and kinks. However, the information generated from the CVs of well-defined surface is important and can be used to get a qualitative estimate of different surface sites on Pt shape-controlled nanoparticles (olla-Gullón et al., S2008). To get a valid correlation between faceting on nanoparticle surface and ORR activity, it is desirable to have quantitative information on various sites present on the surface. The fraction of low-index facets on the nanoparticle surface can be obtained from the specific site-probe reactions involved in the voltammograms of irreversible adsorption of Ge and Bi on {100} and {111} facets, respectively (Clavilier et al., 1988; Gomez et al., 1992; Feliu et al., 1993; Rodriguez et al., 2005; Solla-Gullón et al., 2008). Table 2 presents the relative percentages of estimated low-index sites ({111} and {100}) on shape-controlled Pt nanoparticles (Solla-Gullón et al., 2008; Devivaraprasad et al., 2014). Most importantly, such characterization of the surface structure is conducted in the same experimetal condition (electrolyte, temperature, pressure, etc) as that is used in the reaction for which the electrocatalyst is intended.

**Table 2:** Fractions of {111} and {100} sites obtained using irreversible Bi and Ge adsorption, respectively, for various of shape-controlled Pt nanoparticles.

**Nanoparticles type**	**Bi % {111} sites**	**Ge % {100} sites**
Pt-PC	2 ± 1	21 ± 2
Pt-NC	13 ± 2	52 ± 2
Pt-TD	60 ± 2	14 ± 2
Pt-CO	30 ± 2	45 ± 2

*Reproduced with permission (Devivaraprasad et al., 2014). Copyright 2014, American Chemical Society (ACS)*.

TEM provides the information on the size of the nanoparticles and fraction of various shapes present (shape selectivity) in the bulk of the sample. It also offers the surface atomic arrangement of a specific shape-controlled nanoparticle, which can vary significantly from one particle to another. When the characterization is limited to individual nanoparticles or a selected few particles, the derived conclusions can be obscured and it may not be representative of the average surface structure of the sample. In this case, it is important to analyze a large number of particles to obtain a statistically correct information on the surface. In other words, the available *ex-situ* experimental techniques for the surface characterization of shape-controlled Pt nanoparticles do not emulate the practical electrochemical conditions.

**Figure 6:**
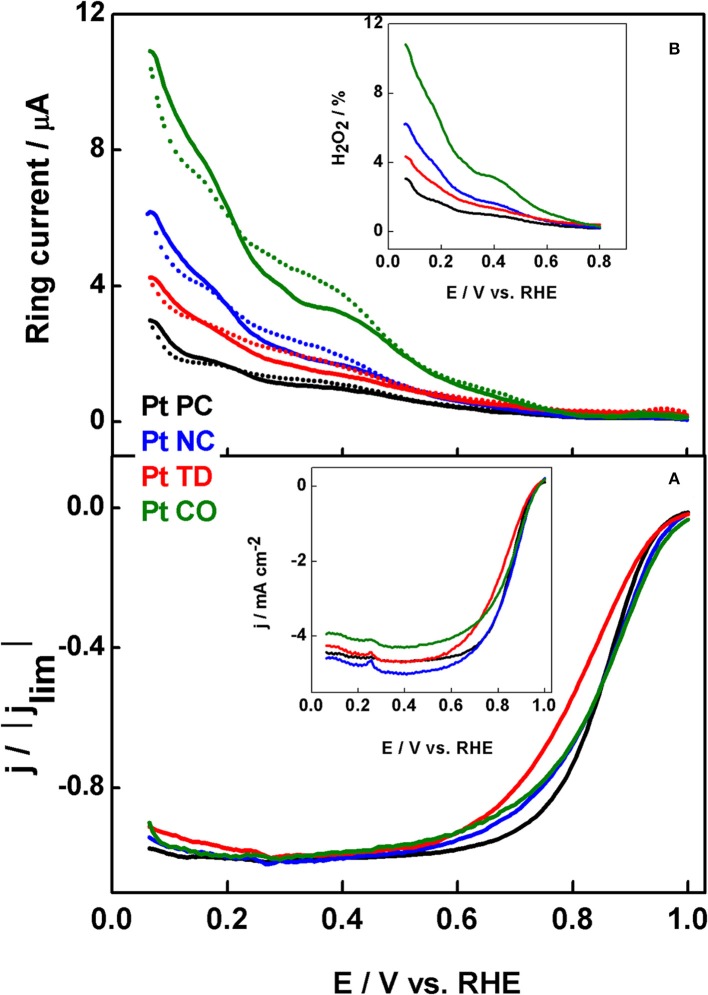
**(A)** ORR voltammograms of Pt-PC, Pt-NC, Pt-TD and Pt-CO nanoparticles recorded in O_2_-saturated 0.5 M H_2_SO_4_ solution at 20 mV s^−1^ with 1,600 rpm. **(B)** H_2_O_2_ oxidation current obtained parallel to the ORR voltammograms; inset to **(B)** shows the fraction of H_2_O_2_ formation during O_2_ reduction. Reproduced with permission (Devivaraprasad et al., 2014). Copyright 2014, American Chemical Society (ACS).

Devivaraprasad et al. (2014) compared the ORR activities and peroxide formation of shape-controlled nanoparticles in acidic and alkaline electrolytes. Figure 6 shows ORR polarization curves of Pt-NC, Pt-CO, and Pt-TD in comparison with that of Pt-PC. In 0.5 M H_2_SO_4_ electrolyte, the activity order is Pt-NC > Pt-CO ≈ Pt-PC > Pt-TD and that in 0.1 M HClO_4_ is Pt-TD > Pt-PC > Pt-NC > Pt-CO. On the other hand, the order of peroxide formation order is Pt-CO > Pt-NC > Pt-TD > Pt-PC and Pt-CO > Pt-NC > Pt-TD ≈ Pt-PC in 0.5 M H_2_SO_4_ and 0.1 M HClO_4_, respectively. At the same time, the activity and peroxide formation trends in 0.1 M NaOH are entirely different. The trends observed are correlated with that of single crystal electrodes reported in the literature. The change in order of ORR activity in the above-mentioned electrolytes is explained on the basis of specific adsorption of anions available on {111}/{100} terrace sites. It is also noticed that the peroxide formation (5–12%) is higher in 0.5 M H_2_SO_4_ and NaOH (strongly adsorbing electrolyte) as compared to that in 0.1 M HClO_4_ (1.5–5%), a weakly adsorbing electrolyte. The variations in the trend of peroxide formation originate from the overlap of ORR with H_upd_ at lower potentials, which inhibits the adsorption of oxygen for reduction. Moreover, there is a particle size effect, wherein a decrease in re-adsorption and reduction of peroxide is observed with increase in particle size of Pt (Devivaraprasad et al., 2014). Sanchez-Sanchez et al. (2010) used the tip generation-substrate collection (TG-SC) mode of scanning electrochemical microscopy and presented the direct observation of different catalytic sites for four shape-controlled Pt NPs catalyzing ORR in two different acid electrolytes (0.1 M HClO_4_ and 0.5 M H_2_SO_4_).

All these studies demonstrate surface structure dependence of ORR. Moreover, they have been able to correlate the results from imaging of shape-controlled nanoparticles and their catalytic activity with that of well-defined single crystal surfaces. These results underline the importance of shape, size and surface structure of nanoparticles in electrocatalysis; moreover, electrocatalytic activity depends strongly on the electrolyte employed for the investigation. Thus, it is important to recall that shape is a necessary prerequisite to define a particular type of surface structure but it is not a sufficient condition; or, shape is an indication of the surface structure. Thus, surface structure and shape are not the same, although they could be correlated, and it is important to differentiate between shape and surface structure at an atomic level.

### Case of Palladium

**Figure 7:**
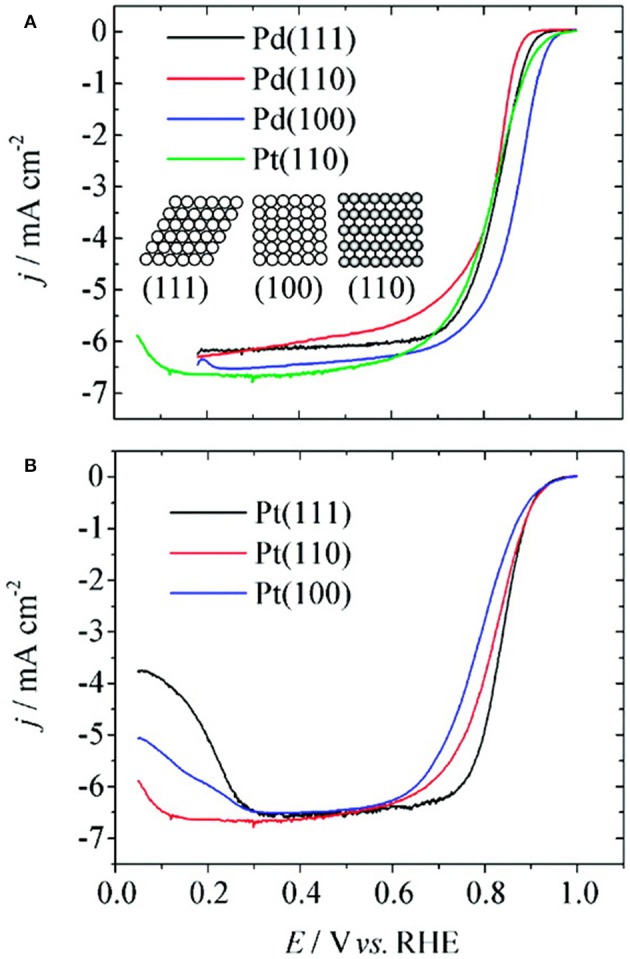
ORR voltammograms of Pd single crystal surfaces **(A)** and Pt single crystal surfaces **(B)** at scan rate of 10 mV s^−1^ in 0.1 M HClO_4_ solution; inset shows the hard sphere model of low index planes of Pd and Pt. Reproduced with permission (Kondo et al., 2009). Copyright 2009, American Chemical Society (ACS).

Investigations on single crystal surfaces of Pd demonstrate that, at 0.9 V (RHE), the specific activity of Pd (100) toward ORR is three times higher compared to that of Pt (110). Moreover, the ORR activity of Pd single crystal surfaces (Figure 7) is in the order of Pd (110) < Pd (111) < Pd (100), opposite to that with respective Pt surfaces in 0.1 M HClO_4_ solution (Kondo et al., 2009). The DFT studies suggest that the order of adsorption energy between O adatom and Pd facets is O-Pd (100) > O-Pd (111) > O-Pd (110) and thus the weak interaction between O adatom and Pd (110) facet is proposed to be the reason for the observed lower activity (Xiao et al., 2009).

Investigation using X-ray scattering were conducted to understand the surface of Pd single crystal under electrochemical conditions and to find active site for ORR. It was reported that there is substantial expansion in the inter layer spacing (5.3%) between d_12_ and d_23_ (d_12_ and d_23_; first and second, second and third, respectively) on Pd (100) surfaces while it is only 1.8% on Pd (111) surfaces in oxygen-saturated solution of 0.1 M HClO_4_. Therefore, the high degree of expansion with Pd (100) compared to Pd (111) may correspond to stronger interaction of oxygen with Pd (100) and hence leads to high activity (Naito et al., 2011). The ORR voltammograms over carbon-supported shape-controlled Pd nanoparticles suggest that there is 10-fold of higher ORR activity with Pd nanocubes compared to that of Pd octahedral in 0.1 M HClO_4_ solution. The *in-situ* production of oxygen-containing species (O_2_, O, OH, and OOH) and their unique interaction with Pd facets were proposed to be the reason for structural dependence of Pd for ORR (Shao, 2011; Shao et al., 2011, 2013). Besides, in strongly adsorbing electrolytes (0.5 M H_2_SO_4_), the same trend in activity (that is higher activity with Pd NC compared to Pd octahedral) was observed; however, there is overall decrease in activity because of strong adsorption of bi(sulfate) anions on the catalyst surface. It was reported that the symmetry between Pd (111) facets and oxygen atoms in bi(sulfate) anions may drastically lower the ORR activity by significantly hindering the adsorption of O_2_ molecules. On the other hand, there was no such facet dependent ORR activity with shape dependent Pd nanoparticles in 0.1 M NaOH solution; all the nanoparticles showed similar activities. This result is different from that of the previous reports of high activity with Pd NC as compared to spherical and cubooctahedral Pd nanoparticles.

**Figure 8:**
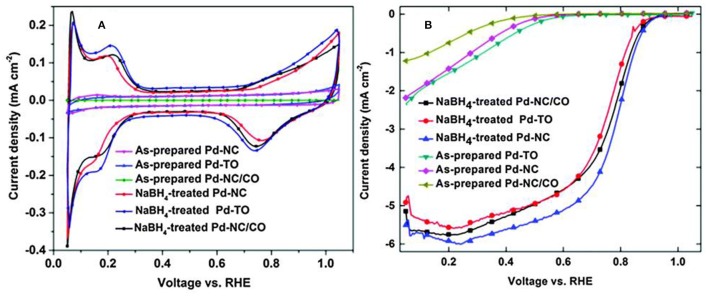
**(A)** CVs, **(B)** ORR voltammograms of shape-controlled Pd nanoparticles (as prepared and that subjected to NaBH_4_ treatment) recorded in 0.1 M HClO_4_ at a scan rate of 20 mV s^−1^. Reproduced with permission (Nalajala et al., 2014). Copyright 2014, Royal Society of Chemistry (RSC).

The electrochemical characteristics of Pd surface after cleaning with NaBH_4_ is shown in Figure 8. Clean precious metal Pd CV features, H_upd_ (0–0.35 V), double layer (0.35–0.7 V) and metal oxidation/reduction (0.7–1.05 V) regions were observed from Figure 8A. The ORR voltammograms in Figure 8B of cleaned shape-controlled Pd nanoparticles suggested that Pd- NC dominant with (100) facets was more active compared to Pd octahedral dominant with (111) facets; these results were in line with that of Pd single crystal surfaces and other reports those were established on shape-controlled Pd nanoparticles. Therefore, this study established a cleaning method based on Pd hydride formation, which can remove the impurities without disturbing the structural integrity of shape-controlled Pd nanoparticles (Nalajala et al., 2016).

Erikson et al. (2016) carried out a comprehensive investigation with carbon-supported Pd-NCs of different sizes (~7, ~10, ~30 nm) and loading from 20 to 50 wt% for ORR in acidic solutions (0.1 M HClO_4_ and 0.5 M H_2_SO_4_). The ORR activity results suggested that the specific activity of Pd-PC dominant with all facets is lower compared to that of Pd-NCs dominant with (100) facets, but decreases with decrease in size of the nanocube. Whereas, the mass activity of Pd-NC increases with decrease in size of the nanocube.

**Figure 9:**
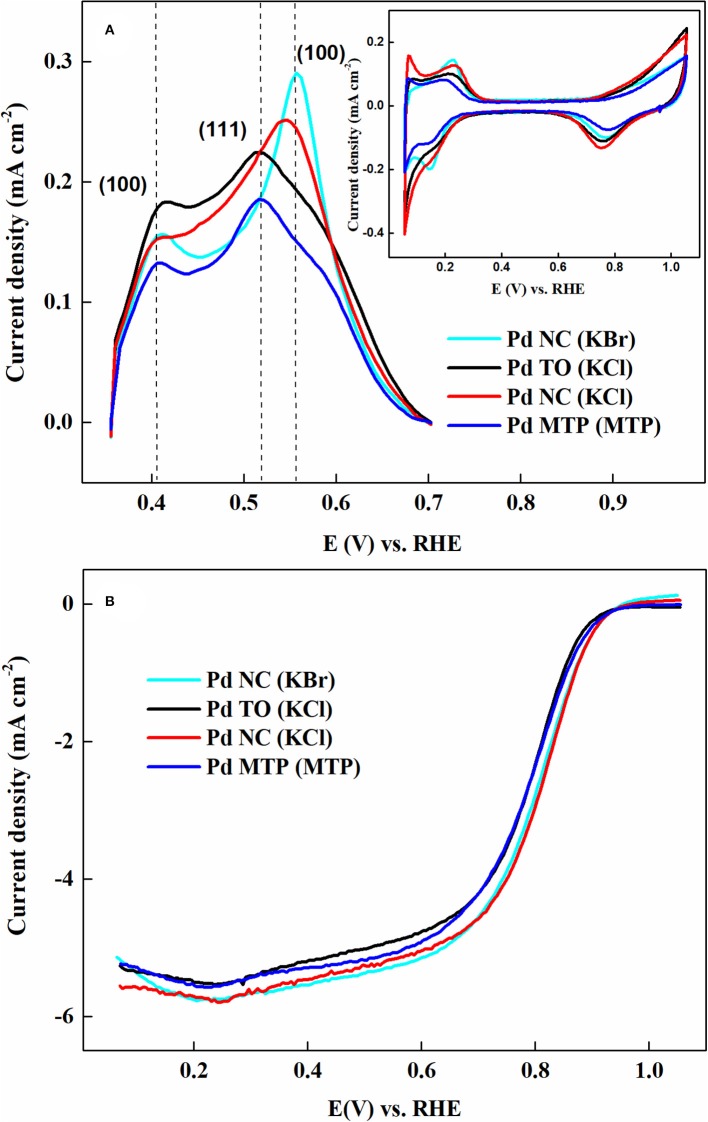
**(A)** Cu stripping voltammograms of shape-controlled Pd nanoparticles recorded at scan rate of 10 mV s^−1^ in an Ar-saturated 0.1 M HClO_4_ contained 12 mM CuSO_4_; inset presents background CVs recorded in the same solution and scan rate but without 12 mM CuSO_4_. **(B)** ORR voltammograms of shape-controlled Pd nanoparticles recorded at scan rate of 20 mV s^−1^ with 1,600 rpm in O_2_-saturated 0.1 M HClO_4_ solution. Reproduced and modified with permission (Nalajala et al., 2016). Copyright 2016, Institute of Physics (IOP).

Figure 9 demonstrates the copper stripping voltammograms and their corresponding background voltammograms (inset to Figure 9A). Conventionally, the surface area is measured from the charge associated with CO stripping voltammetry and H_upd_ region of the voltammogram. In this context, the under potential deposition of metal adatoms (for e.g., Cu) above their reversible potential on foreign metal substrates/surfaces is employed in order to explore the surface area of catalyst materials. Due to distinct adsorption energies of Cu, it is also possible to probe and identify the dominant crystallographic facets, which are present on the surface of catalyst (Pronkin et al., 2007). The appearance of peaks at different potentials may be correlated to the binding energies between the adatoms (for e.g., Cu) and the atoms of the substrate/surface. Moreover, using the Cu stripping method, one can explore the surface composition of the electrocatalyst. The facet dominance of Pd nanoparticles are clearly evident from the Cu stripping voltammograms; intense peaks observed at 0.41 V and 0.56 V were assigned to (100) facets of Pd NC samples those were prepared by both KCl and KBr as capping agents. Besides, the intense peak at 0.51 V, corresponding to (111) facet, was quite dominant with truncated octahedral and MTP shapes of Pd. The ORR voltammograms (Figure 9B) of these samples recorded in O_2_-saturated 0.1 M HClO_4_ solution suggested that the trend in half-wave potentials is in accordance to the activity that was reported on Pd single crystal surfaces; (100) facet is more active compared to (111) facet. At the same time, features of Pd were not observed for the nanoparticles synthesized with I^−^ ion as capping agent, demonstrating that NaBH_4_ could not clean adsorbed I^−^ impurities; thus, one cannot observe electrocatalytic activity for ORR.

According to Sabatier's principle, there should be an optimum binding between reactants, intermediates, products and metal surface for efficient catalytic and electrocatalytic reactions. In this context, recently, Wang et al. (2019) proposed a general and powerful strategy that provides the tuning of intrinsic surface strain in the metal nanosheets for electrocatalytic properties; for instance, ORR in alkaline and acid solutions. They found that the specific activity (SA) in 0.1 M HClO_4_ at 0.95 V for 3-ML, 5-ML, 8-ML was 10, 14, 5 times, while in 0.1 M KOH was 15, 18, and 2 times higher than that of Pd nanoparticles, respectively. Through DFT studies, they established a relationship between thickness of the slab and compressive strain that causes ORR activity. It is found that 0.35, 0.92, and 2.50% compressive strain was obtained for 8-ML, 5-ML, and 3-ML, respectively. In general, the unstrained surface binds oxygen very strongly, which limits the ORR by slow oxidative desorption of hydroxides. On the other hand, with decreasing slab thickness, providing more compressive stains enables weak binding of O^*^ and thus provide better ORR kinetics. Therefore, in accordance with Sabatier's principle, the optimum thickness of slab (5-ML) would be able to exhibit much better ORR activities in both alkaline and acidic solutions. In the literature it is reported that PdH_0_ nanocubes can exhibit comparable activity as that of commercial Pt/C. Moreover, PdH_0.7_ exhibits mass activity ~3.75 times better than that of PdH_0_ (Lu et al., 2017).

Literature is replete with shape-dependent ORR in acidic solutions (HClO_4_, H_2_SO_4_) and alkaline solutions (NaOH, KOH) for fuel cell applications. ORR in ionic liquids (ILs) is of technological and scientific importance in lithium-air/lithium-oxygen batteries. For instance, rechargeable lithium-air batteries provide 3–5 times better gravimetric energy density compared to conventional Li-ion batteries. However, poor electrolyte stability in the presence of ORR intermediates, such as superoxide anion radicals (${\text{O}}_{2}^{*}$), is one of the major challenges in the development of Li-air/Li-O_2_ batteries. Therefore, studies on ORR in ILs using different metal surfaces and nanoparticles received significant attention recently (Laoire et al., 2010; Ernst et al., 2011). In general, electrolytes based on ILs are promising because of their low volatility, low toxicity, high ionic conductivity, thermal, chemical and electrochemical stability (Schwenke et al., 2015). In this context, Evans et al. (2004) pursued a comprehensive investigation over electrogeneration of stable superoxide ions at different material surfaces such as platinum, gold, and glassy carbon electrode in various room temperature quaternary ammonium ILs and quaternary phosphonium ILs. Lu et al. (2011) carried out remarkable studies on polycrystalline Pd, Au, Pt, Ru, and glassy-carbon via rotating disk electrodes (RDE) measurements in non-aqueous 0.1 M LiClO_4_ in 1,2-dimethoxyethane electrolyte and found that the volcano type Li^+^-ORR activity is in the order of Pd > Pt > Ru ≈ Au > GC. Moreover, it is established that the obtained ORR activity trend is in good agreement with the discharge voltage of Li-O_2_ cells those were made of nanoparticle catalysts (Lu et al., 2011). Recently, the shape-dependent ORR activity of Pd in IL, 1-butyl-1-methylpyrrolidinium bis(trifluoromethylsulfonyl)imide ([Bmpy][NTf2]), was reported (Tang et al., 2016). The prepared nanoparticles were immobilized on gas permeable membrane for measuring the ORR activity in the afore-mentioned ionic liquid. It was observed that rhombic dodecahedral Pd nanoparticles dominant with high energy facet (110) possess better catalytic activity for oxygen reduction compared to that of cubic Pd with dominant (100) facets (Tang et al., 2016). The literature on shape-dependent ORR in ILs is very limited and more efforts are required to understand the facet-dependent ORR kinetics in ILs (Girishkumar et al., 2010; Wang et al., 2014).

## Stability of Metal Nanoparticles Under Electrochemical Conditions

Various researchers investigated stability of Pt single crystal electrodes during the potential cycling in the electrochemical environment. Thus, Clavilier and Armand (1986) established the dissolution rates over a range of potentials (Yamamoto et al., 1979; Clavilier and Armand, 1986; Marković et al., 1988, 1991; Wagner and Ross, 1988; Rodes et al., 1990; Kolb, 1995). It was concluded that the rate of dissolution is slow in the potential range of 0.06 to 0.8 V with any given Pt surface and it is unlikely to occur.

In case of Pt(111) surface, the so-called characteristic “butterfly features” observed in the potential range of 0.4–0.6 V in acidic electrolyte and other typical Pt features observed in alkaline electrolyte remains undisturbed, only if the upper potential limit is restricted to ≤ 0.8 V. These features of the reversible adsorption of hydrogen and anions in the H_upd_ region correspond to well-ordered (111) terrace sites (Kolb, 1995). In case of Pt(100) surface, significant changes in the voltammetric features were noticed even with potential cycling in the lower ranges. Thus, the stability of (100) surfaces is inferior to that of (111) surfaces (Clavilier and Armand, 1986; Wagner and Ross, 1988; Kolb, 1995). Investigations on single crystal surfaces ({100}; {111}; {110}), when subjected to potential cycling above 1.0 V, offer evidence for dissolution and reconstruction and it is influenced by the adsorbates and temperature (Clavilier and Armand, 1986; Wagner and Ross, 1988; Kolb, 1995).

Lopes et al. (2016) developed a unique method that utilized a stationary probe coupled to Inductively Coupled Plasma Mass Spectrometry (ICP-MS), combined with a rotating Pt(hkl)-disk electrode (RDE), to study the role of surface geometry on the stability of surface atoms. It enabled “atom-by-atom” detection of the adsorbate-induced dissolution of Pt in acidic and alkaline environments. This study demonstrated that the structure-stability relationships established for platinum single crystals can be used as a foundation for understanding the stability of polycrystalline Pt electrodes and Pt nanoparticles. It was shown that the overall dissolution rates were driven by a synergy between electrochemical (potential induced oxide formation) and chemical corrosion (thermodynamic driving force for Pt complexation). It was also shown that the dissolution dynamics were strongly affected by the nature of the electrochemical reaction; thus, continuous dissolution occurs during the oxygen evolution reaction (OER), limited dissolution during CO oxidation reaction, and no dissolution during the ORR. Reconstruction and dissolution of Pt surface are evidenced by the new voltammetric features at specific potentials and by the presence of Pt^2+^ ions in the electrolyte, respectively. But, long-term electrochemical investigations on extended single crystal surfaces are limited.

With conventional polycrystalline nanoparticles, all the low-index crystallographic orientations feature on the surface (no preferential dominance); therefore, investigating the extent of reconstruction and dissolution is a daunting task. Nevertheless, the reconstruction of nanoparticles is investigated using high pressure scanning tunneling microscopy (HP-STM), ambient pressure X-ray photoelectron spectroscopy (AP-XPS), *in situ* XRD, and TEM (Arenz et al., 2003; Garcia-Araez et al., 2005). With the change in temperature, pressure and potential, metal nanoparticles are susceptible to surface structural changes. The stability of Pt nanoparticles is also affected by pH, potential and presence of adsorbates (Kinoshita et al., 1973; Komanicky et al., 2006; Wang et al., 2006; Yasuda et al., 2006; Tang et al., 2010a,b; Matsumoto et al., 2011; Sugawara et al., 2011, 2012; Kongkanand and Ziegelbauer, 2012; Topalov et al., 2012, 2014a,b; Ahluwalia et al., 2013; Xing et al., 2013; Cherevko et al., 2014; Arán-Ais et al., 2018). Unfortunately, conclusions derived from spectroscopic analytical tools and measurements carried out under ultra-high vacuum conditions (UHV) with single crystal surfaces cannot be directly implied with nanoparticle surfaces. At this juncture, work on shape-controlled Pt nanoparticles has, to some extent, bridged the gap between the single crystal electrodes on one hand and Pt-based fuel cell electrocatalysts on the other hand.

Recently, Identical Location Transmission Electron Microscopy (IL-TEM) was used for the investigation of shape-controlled nanoparticles during electrochemical cycling, a process routinely followed in the activation of catalyst surface (Arán-Ais et al., 2015a). They reported significant changes in the shape and morphology of hexagonal nanoparticles and particle migration on potential cycling to 1.3 V (Arán-Ais et al., 2015b).

**Figure 10:**
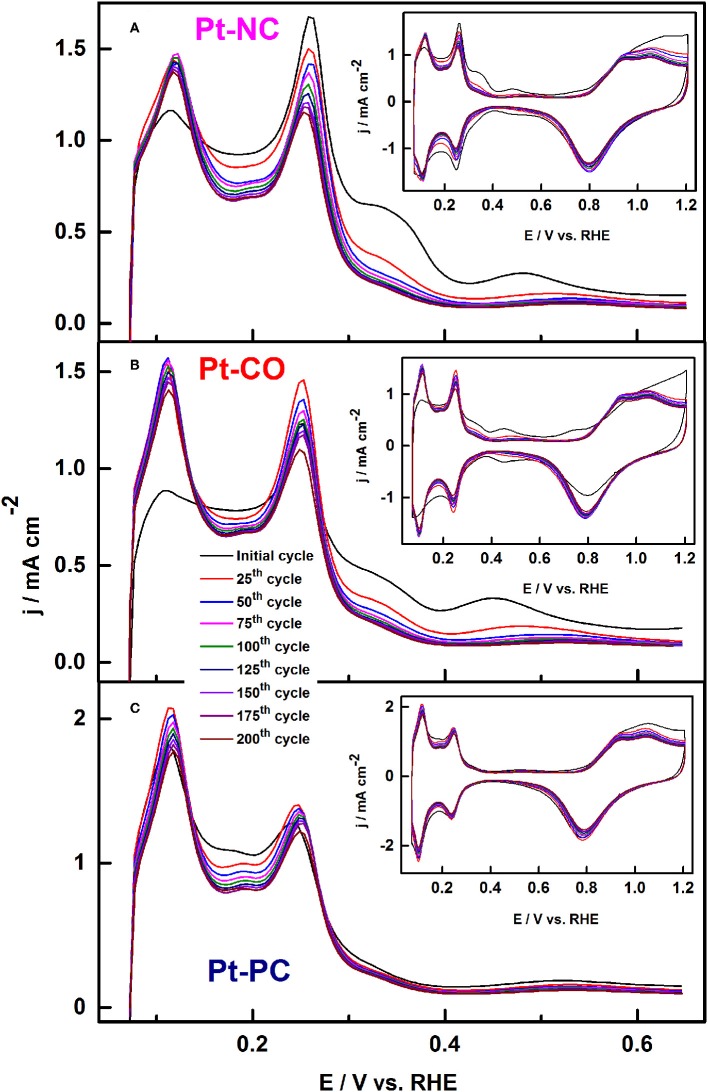
Potential cycling of **(A)** Pt-NC, **(B)** Pt-CO, and **(C)** Pt-PC nanoparticles upto 1.2 V in argon-saturated 0.5 M H_2_SO_4_ electrolytes at a scan rate of 50 mV s^−1^ (every 25th cycle upto 200 cycles). Inset shows the respective complete CVs up to 1.2 V. Reproduced with permission (Devivaraprasad et al., 2016). Copyright 2016, Royal Society of Chemistry (RSC).

**Figure 11:**
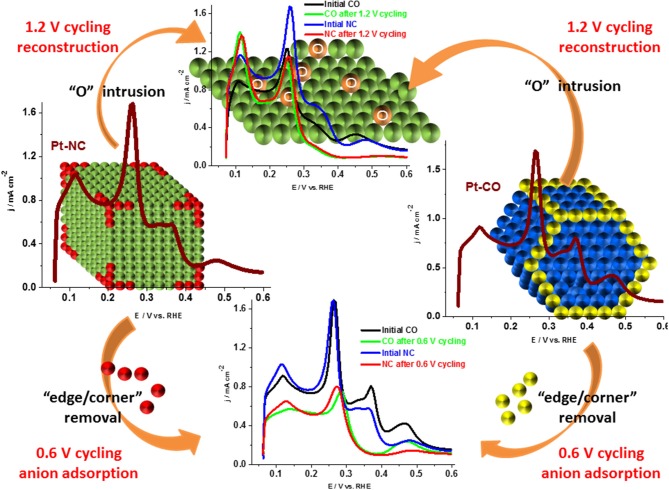
Schematic showing Pt dissolution and reconstruction along with the corresponding voltammetric profiles in the Hupd region. Reproduced with permission (Devivaraprasad et al., 2016). Copyright 2016, Royal Society of Chemistry (RSC).

Similar results were reported recently by Devivaraprasad et al. (2016) and Arán-Ais et al. (2015b) in the investigation of stability and surface structure of clean shape-controlled nanoparticles in 0.5 H_2_SO_4_ electrolyte. It was observed that Pt-NC and Pt-CO reconstruct to Pt-PC on potential excursion to 1.0 and 1.2 V. Such changes to the shape of the nanoparticle is correlated with the rise in peak features at potential 0.125 and 0.27 V in the H_upd_ region originating from the {110} sites and {100} steps sites, at the expense of {100} and {111} terrace sites (Figure 10). Unlike expected, the reduction in H_upd_ area is negligible on potential excursion of the electrode to 1 and 1.2 V. This is because of the protective oxide surface on Pt, which hinders further dissolution. When potential limit is restricted to 0.6 or 0.8 V, there is a significant decrease in the H_upd_ area of both Pt-PC and shape-controlled Pt nanoparticles as presented in Figure 11. Such reduction in H_upd_ area is due to adsorption and subsequent accumulation of electrolyte anions. The recovery of the H_upd_ charge on cycling to potential above 1 V supports the above-mentioned conclusion. Thus, the adsorbed anions were substituted with oxygenated species on Pt, which subsequently gets reduced at lower potentials. Amongst different shape-controlled Pt nanoparticles, Pt-NCs dissolved at a faster rate than Pt-CO or Pt-PC. The HRTEM images demonstrated the loss of {111} and {100} terraces and led to spherical particles of comparable size, conforming with the observation from the voltammograms (Devivaraprasad et al., 2016).

Some of these findings evidently shed light on various aspects of the stability of metal nanoparticles, and most importantly, emphasize the potential range to be considered in the investigation of shape-dependent electrochemical reactions. Thus, one has to be extremely careful while deriving conclusion in the investigations on shape-controlled nanoparticles, particularly with reactions involving high potentials, such as that with ORR. It is well-established in the literature that the stability of Pd nanoparticles is poorer than that of Pt nanoparticles, and the readers are suggested to refer previous references and discussion on such aspects is beyond the scope of this review (Meier et al., 2005; Wells et al., 2009; Singh et al., 2013; Mittermeier et al., 2015; Zadick et al., 2016).

## Non-recoverable ESA Losses With Halide Ion Adsorption and Active Surface Recovery Methods of Shape-Controlled Pt Nanoparticles

The adsorption processes at platinum electrodes are known to complicate the kinetics of surface-sensitive electrochemical reactions. Thus, ORR was investigated at platinum electrode surfaces in the presence of various cations (Na^+^, K^+^, etc.) (Okada et al., 1999; Durst et al., 2012), anions (${\text{CIO}}_{4}^{-}$, ${\text{HSO}}_{4}^{-2}$, etc.) (Hsueh et al., 1983), halides (Cl^−^ and Br^−^) (and Marković et al., 1999; Schmidt et al., 2001; Stamenkovic et al., 2001) and other impurities (${\text{P}}_{4}^{3-}$, ${\text{NH}}_{4}^{+}$, CO, SO_2_, H_2_S, etc.) (Macia et al., 2004; Stamenkovic et al., 2005; Halseid et al., 2008; He et al., 2010; Ong et al., 2013; Rahul et al., 2015; Briega-Martos et al., 2018). Therefore, strongly adsorbing anions/cations/impurities/*in situ* generated adsorbed species on the catalyst surface interfere with the investigation of dissolution and reconstruction of nanoparticles, making it is hard to investigate the stability, origin of the loss of ESA, and catalytic activity of nanoparticles.

Influence of halides ions adsorbed on single crystal electrodes of Pt was extensively reported by various groups (Solomun et al., 1984; Salaita et al., 1986; Shu and Bruckenstein, 1991; Vogel and Baltruschat, 1991; Oelgeklaus et al., 1994; Albers et al., 1995; DeSimone and Breen, 1995; Abruna et al., 1998; Arenz et al., 2003; Garcia-Araez et al., 2004, 2005, 2006a,b). AES/LEED investigations lead to the conclusion that the Cl^−^ ion adsorption occurs on Pt (100) and Pt (111) surfaces at two different potentials, i.e., in the H_upd_ and OH adsorption regions, respectively (Arruda et al., 2008). The Pt (100) surface was shown to be affected by Cl^−^ adsorption at a lower potential than Pt (111) surface, suggesting higher vulnerability of the former to chloride ions (Arruda et al., 2008).

In the process of translating the understanding of fundamental studies carried out at single crystal surfaces to the nanoparticle surfaces, the effect of halide ions (I^−^, Br^−^, Cl^−^) on H_ads/des_ and reconstruction of shape-controlled Pt nanoparticles (Pt-NCs and Pt-COs) and Pt-PCs were investigated in 0.5 M H_2_SO_4_ electrolyte (Devivaraprasad et al., 2017). Characteristic facet-dependent I^−^, Br^−^, Cl^−^ adsorption/desorption features were observed and the adsorption strength of halides was in the order of I^−^ > Br^−^ > Cl^−^; thus, the H_upd_ area of Pt decreased significantly with the addition of I^−^ ions to 0.5 M H_2_SO_4_ electrolyte. With Br^−^ and Cl^−^ ions, Pt exhibited a concurrent H_ads/des_/${\text{Br}}_{\text{ads}/\text{des}}^{-}$ /${\text{Cl}}_{\text{ads}/\text{des}}^{-}$ features in the H_upd_ region (0.06–0.4 V). As discussed in section Stability of Metal Nanoparticles Under Electrochemical Conditions on the effect of potential cycling in pure electrolytes, it is observed that the shape-controlled Pt nanoparticles undergo reconstruction in the presence of halide ions as well. Thus, potential cycling in the range of 0.06–1.4 or 1.6 V causes the conversion of ordered terraces to disordered sites, resulting in eventual Pt-PC voltammetric features (Devivaraprasad et al., 2017).

**Figure 12:**
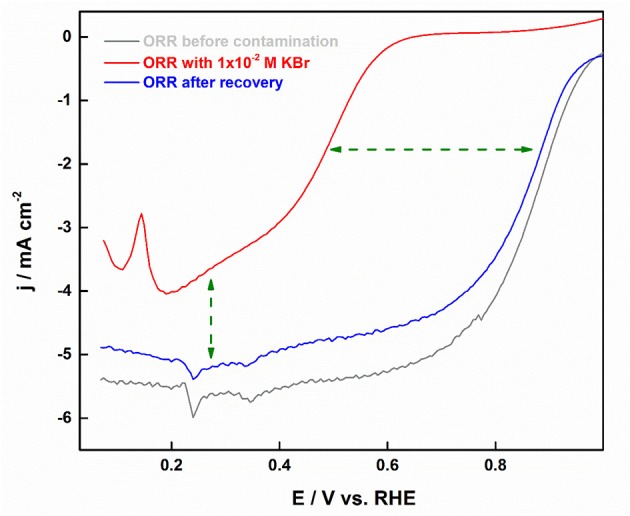
ORR polarization curves with Pt-CO in oxygen-saturated 0.5 M H_2_SO_4_ in relation to that intentionally contaminated with 10^−3^ M Br^−^ ions. Reproduced with permission (Devivaraprasad et al., 2017). Copyright 2017, Journal of the Electrochemical Society (ECS).

An *in situ* potentiostatic method was devised for the removal of absorbed halide ions from the Pt surface, wherein the working electrode was immersed in 0.1 M NaOH solution at ~0.03 V. This method was proved to be effective enough to recover the active surface area without altering the facet-structure of shape-controlled nanoparticles. Figure 12 shows an *in-situ* cleaning procedure employed in recovering the ORR activity.

Recently, Unnikrishnan et al. (2018), also reported the deleterious effects of Cl^−^ ions on introducing to anode and cathode sides of a PEMFC. At an operating voltage of 0.6 V and due to the presence of bare minimum amount of Cl^−^ ions (100 ppm) in the feed stream, a performance loss of ~94% with the anode and ~82% with the cathode were observed (Unnikrishnan et al., 2018). While operating at higher current densities, a significant loss can be avoided due to the recovery processes (removal of Cl^−^ species adsorbed on the platinum surface) happening at the anode side with the help of protons and on the cathode side with help of the hydroxyl ions (Unnikrishnan et al., 2018).

Adsorption of anions (sulfates and halides) is well-known to poison the catalyst surface and to retard the catalytic activity. Adsorbed impurities negatively impact the performance of fuel-cells, batteries and electrolyzers and the activity of a surface depends strongly on the structure and cleanliness of the surface. These finding have important implications in recovering the active catalyst surface contaminated with impurities. Also, some of the methods discussed here may be a stepping stone in the direction of making fuel cells with long-term durability and sustained catalytic activity.

## Conclusions and Future Prospect

Investigations on well-defined single crystal electrode surfaces have established the facet dependence of various electrochemical reactions on precious metal surfaces (Pt and Pd). The adsorption free-energy on various facets decides the electrochemical activity. In an attempt to take advantage of the facet dependence of electrochemical reactions, various shape-controlled nanoparticles were synthesized using surfactants/capping agents/stabilizers etc. But, after the synthesis, it is essential to remove the surfactants from the surface of shape-controlled metal nanoparticles for enhanced electrocatalytic activity—indeed a daunting task. Although various cleaning protocols have been proposed and proved to be efficient, it is important to understand that, most of these cleaning procedures are specific to the removal of individual surfactants or stabilizer or synthesis protocol employed and therefore the search for universal surfactant removal procedure is still underway.

Moreover, the facet dependent ORR activity of shape-controlled nanoparticles is not as distinctive as that expected from that of single crystal surfaces. This is due to the exposure of a mix of almost all the low index crystallographic orientations on the nanoparticle surface along with surface defects, steps, kinks, edges and corners. However, methods based on Bi and Ge adsorption do provide means to estimate fraction of low-index sites and compare their ORR activities with that of well-defined surfaces; more work needs to be done in this area for estimation of contributions from other facets of shape-controlled nanoparticles. But this whole exercise has led to more information on long-term performance issues of polycrystalline Pt. Investigations on shape-controlled nanoparticles not only bridge the gap between fundamental studies and practical electrocatalysts employed, but also provide significant information on reconstruction, dissolution, specific adsorption of anions on the particle surfaces and recovery of electrocatalytic activity. Perhaps, nanoparticles with high-index sites stable under the fuel cell relevant potentials would be an ideal solution for high ORR activity. Designing such highly active and stable electrocatalysts, devoid of impurities, is still a work in progress.

## Author Contributions

All authors listed have made a substantial, direct and intellectual contribution to the work, and approved it for publication.

### Conflict of Interest

The authors declare that the research was conducted in the absence of any commercial or financial relationships that could be construed as a potential conflict of interest.
